# Durable Response to Sintilimab and Chidamide in a Patient With Pegaspargase- and Immunotherapy-Resistant NK/T-Cell Lymphoma: Case Report and Literature Review

**DOI:** 10.3389/fonc.2020.608304

**Published:** 2020-12-11

**Authors:** Zheng Yan, Shuna Yao, Yanyan Liu, Jianbo Zhang, Peng Li, Haiying Wang, Junfeng Chu, Shuang Zhao, Zhihua Yao

**Affiliations:** ^1^ Department of Internal Medicine, Affiliated Cancer Hospital of Zhengzhou University and Henan Cancer Hospital, Zhengzhou, China; ^2^ Department of Pathology, Affiliated Cancer Hospital of Zhengzhou University and Henan Cancer Hospital, Zhengzhou, China; ^3^ The PET-CT Center of Henan Province, Affiliated Cancer Hospital of Zhengzhou University and Henan Cancer Hospital, Zhengzhou, China

**Keywords:** NK/T-cell lymphoma, immunotherapy, chidamide, immunotherapy resistance, sintilimab, case report

## Abstract

The prognosis of patients with relapsed/refractory NK/T-cell lymphoma (NKTCL) is dismal. Immunotherapy has showed encouraging anti-tumor activity in patients with asparaginase-resistant NKTCL; however, only a portion of patients benefit and the median response duration is rather short. Treatment strategies have not been identified for immunotherapy-resistant NKTCL. We describe a patient with primary cutaneous NKTCL experienced disease progression after pegaspargase-based chemotherapy and PD-1 inhibitor (sintilimab)-based immunotherapy. Following a combined treatment of sintilimab and the HDAC inhibitor chidamide, the patient achieved a durable complete molecular response with mild toxicity. This case indicates that the combination of PD-1 inhibitor and HDAC inhibitor might be a treatment choice for immunotherapy-resistant NKTCL.

## Background

NK/T-cell lymphoma (NKTCL) is a rare and aggressive hematological malignancy. It mainly involves the upper aerodigestive tract, less commonly the skin, soft tissue, and gastrointestinal tract (1). Most patients with early-stage NKTCL can be cured with radiotherapy or combined radio-chemotherapy; however, patients with an advanced form of the disease have a dismal prognosis with a median survival of several months (2, 3). Historically, NKTCL responded poorly to conventional cytotoxic drugs probably because of inherent multidrug resistance; while it was sensitive to L-asparaginase (or pegaspargase) due to the unique anti-tumor mechanism for asparaginase and the fatal weakness of NKTCL cells. All normal body cells can produce asparagine by asparagine synthetase, while NKTCL cells are unable to synthesize the amino acid. Asparaginase therapy depletes serum asparagine. When the host serum asparagine is depleted by asparaginase, protein synthesis in NKTCL cells stops, leading to cancer cell death (4). Asparaginase alone or in combination with different cytotoxic drugs has showed good immediate efficacy in NKTCL treatment (5). However, disease relapse frequently occurs due to acquired drug resistance to asparaginase. Nowadays, there are limited treatment options for asparaginase-resistant NKTCL.

Recently, anti-PD-1/PD-L1 immunotherapy constitutes a new treatment option for relapsed/refractory (r/r) NKTCL. The PD-1 inhibitors pembrolizumab and sintilimab, as well as the PD-L1 inhibitor avelumab, have yielded encouraging effects in several case series reports and small sample-sized clinical trials (6); however, only a small portion of patients benefit, reflecting common primary and/or acquired resistance to immunotherapy and less durable response. Therefore, it is necessary to identify and evaluate novel treatment strategies for immunotherapy-resistant NKTCL. In this study, we reported a case with both pegaspargase- and immunotherapy-resistant NKTCL that achieved durable response to a combined treatment of sintilimab with the histone deacetylase inhibitor (HDACi) chidamide.

## Case Presentation

**Figure 1:**
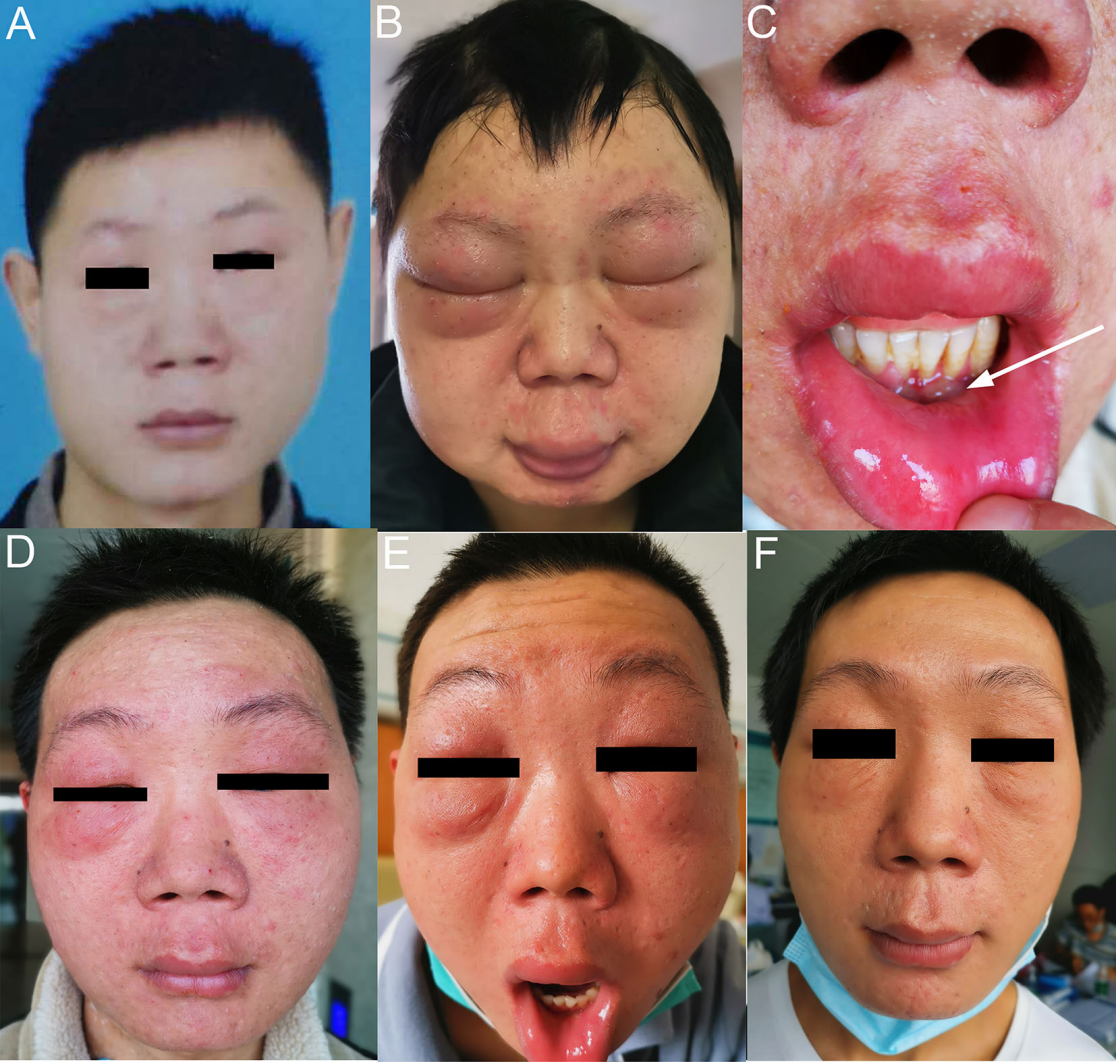
Facial features of the patient with NKTCL. **(A)** Two years before diagnosis. **(B)** At the time of diagnosis of NKTCL. **(C)** Mucosal ulcer in the inner lower lip at diagnosis. **(D)** After completing 3 cycles of P-GemOx regimen chemotherapy. **(E)** After one cycle of sintilimab and decitabine combination treatment. **(F)** After four cycles of sintilimab and chidamide combination treatment.

**Figure 2:**
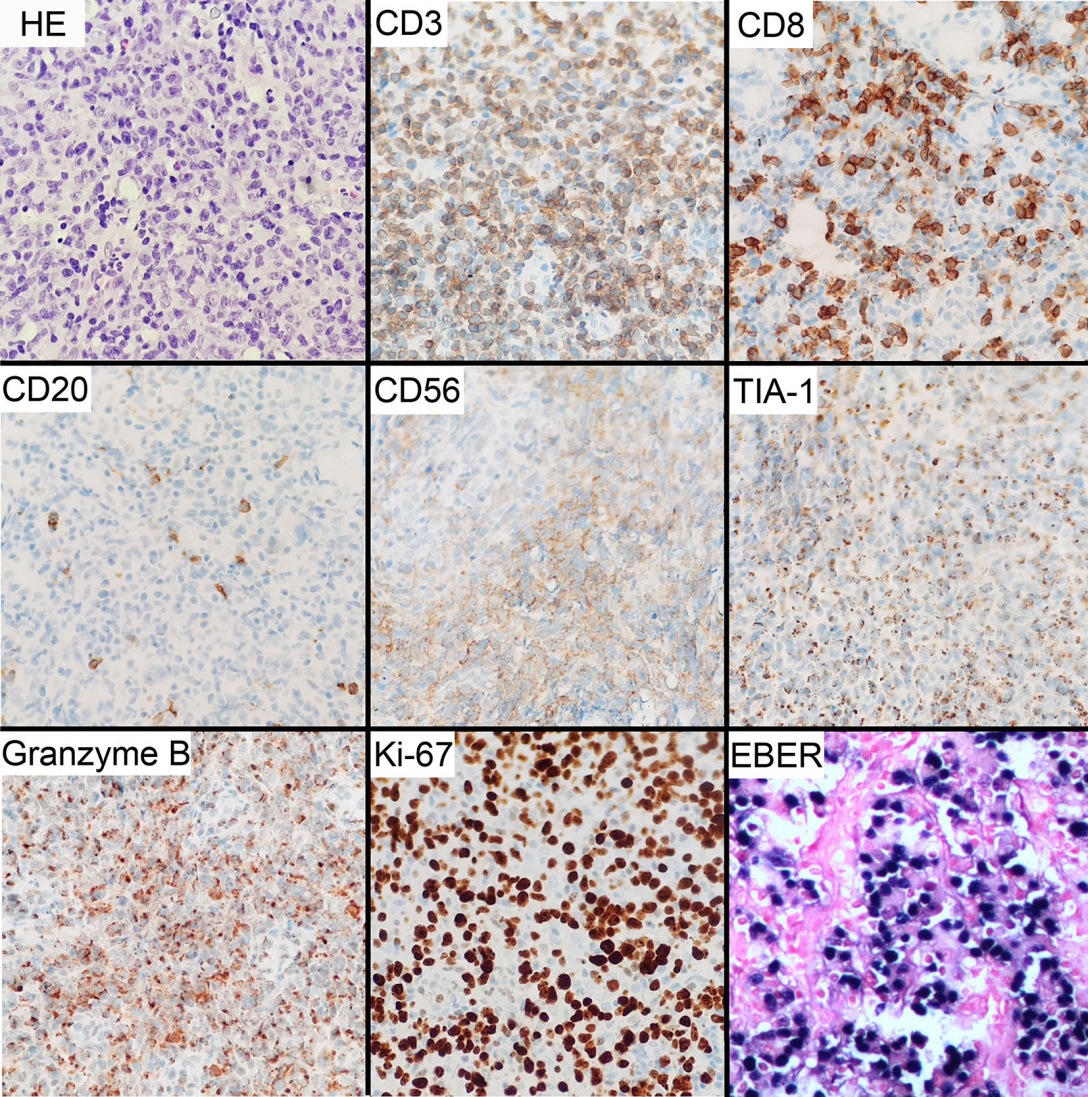
Microscopic and immunohistochemical features of NKTCL tumor. Histological examination of H&E-stained tissues shows infiltration of medium-sized cells with irregular nuclei and inconspicuous nucleoli. Immunohistochemical staining shows positive CD3, CD56, TIA-1, and granzyme B, focally positive CD8, and negative CD20. Ki-67 proliferation index was about 90%. In situ hybridization for EBV-encoded RNA (EBER) reveals positive reaction. Original manifestation 400×.

**Figure 3:**
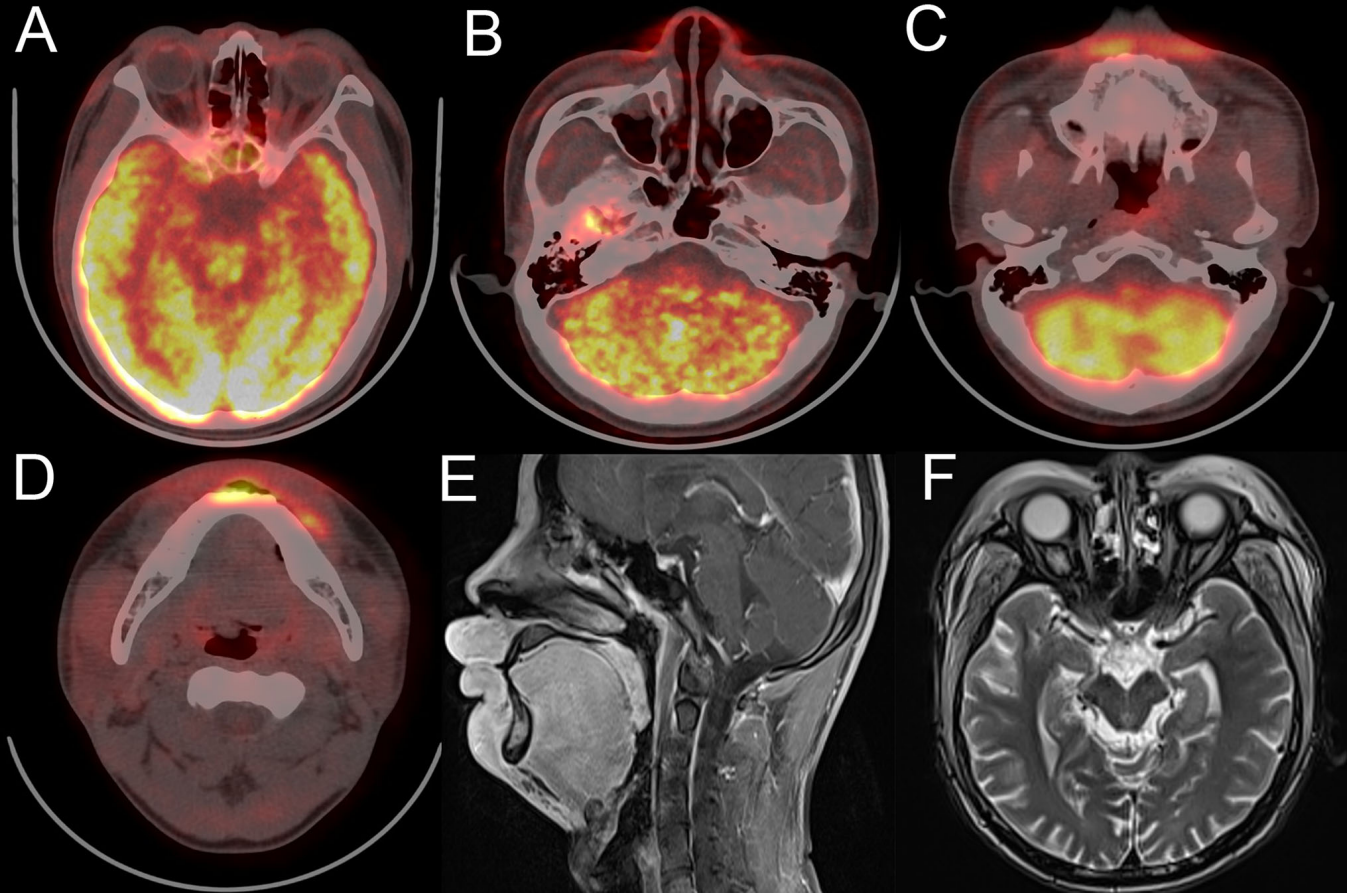
Face imaging features of the patient at diagnosis. **(A–D)** PET/CT shows thickened and FDG-avid midline facial skin. **(E, F)** MRI sagittal plane and horizontal views show thickened facial skin and hyperintensity on T2-weighted imaging.

At his first visit, a 24-year-old young man stated that two years ago his face, especially eyelids, was slightly red and swollen without any discomfort (**Figure 1A**). He had no notable past and family medical history. Since then, his facial swelling has persisted without obvious changes and medical intervention, but in recent two months the face swelling was rapidly aggravated with fever. He was admitted to our hospital in October 2019 with intermittent fever in the absence of weight loss and night sweat. The maximum body temperature was 38.3°C. Physical examination revealed that his whole face was red and swollen with a mucosal ulcer in the inner lower lip (**Figures 1B, C**). Laboratory examination revealed an elevated lactate dehydrogenase level of 319 U/L and erythrocyte sedimentation rate of 39 mm/h. Plasma EBV-DNA titer was 2.11 × 10^4^ copies/ml. Other laboratory parameters were normal. Biopsy from the oral ulcer showed acute and chronic mucosal inflammation with infiltration of medium-sized atypical lymphoid cells. The infiltrating lymphocytes were positive for CD3, CD56, TIA-1, and Granzyme B, negative for CD20 and CD30, and focally positive for CD8. Ki-67 was positive in 90% cancer cells. *In situ* hybridization revealed EBV infection in the majority of neoplastic cells (**Figure 2**). The diagnosis of NKTCL was made based on the morphology, immunohistochemistry, and EBV status. Positron emission tomography-computed tomography (PET/CT) (**Figures 3A–D**) and magnetic resonance imaging (MRI) (**Figures 3E, F**) showed that the facial soft tissues, eyelids, and lips were swollen with hypermetabolic activity (SUVmax 10.3). Bone marrow smear and flow cytometry analysis were negative.

From November 13, 2019 to December 27, 2019, the patient was given three cycles of P-GemOx regimen (pegaspargase, gemcitabine, and oxaliplatin) chemotherapy. After two cycles of treatment, his facial swelling alleviated, fever disappeared, and the plasma EBV-DNA titer reduced to an undetectable level. However, there was no further improvement for the facial swelling following the third cycle of chemotherapy (**Figure 1D**) and the plasma EBV-DNA titer came back and was increased to 1.15 × 10^3^ copies/ml, indicating the development of acquired resistance to pegaspargase-based chemotherapy.

On January 15, 2020, the patient was treated with sintilimab (200 mg) as second-line treatment. In a prior study, decitabine, a DNA methyltransferase inhibitor, was showed to increase the efficacy of PD-1 blockade immunotherapy in patients with Hodgkin lymphoma (7). Considering only a short progression-free survival (PFS) in the trials of immunotherapy in r/r NKTCL patients (**Table 1**), a priming treatment with decitabine was administered to the patient in addition to sintilimab. Unexpectedly, the patient’s facial swelling was slightly aggravated (**Figure 1E**), accompanied by the reappearance of fever, and the plasma EBV-DNA titer was increased to 7.02 × 10^3^ copies/ml. Though the face swelling was not a definite indicator of drug efficacy, the reappearing fever and elevated plasma EBV-DNA titer strongly indicate the disease out of control.

**Table 1:** Reports of r/r NKTCL treated with immunotherapy.

Author	Study	No.	Treatment	Response	PFS
Tao et al. (8)	Phase 2	28	Sintilimab	19 (CR + PR)	–
Kim et al. (9)	Phase 2	21	Avelumab	5 CR, 3 PR	2.7 m (median)
Kim et al. (10)	Retrospective	14	Pembrolizumab	5 CR, 1 PR	–
Kwong et al. (11)	Case series	7	Pembrolizumab	5 CR, 2 PR	–
Li et al. (12)	Case series	7	pembrolizumab	2 CR, 2 PR	4.8 m (median)
Chan et al. (13)	Case series	3	Nivolumab	1 CR	–
Klee et al. (14)	Case report^a^	1	Pembrolizumab + radiotherapy	CR	>2 y
Kim et al. (15)	Case report	1	Pembrolizumab + haploidentical HSCT	CR	>6 m
Asif et al. (16)	Case report	1	Pembrolizumab + radiotherapy	CR	4 m
Lai et al. (17)	Case report	1	Pembrolizumab	CR^b^	>8 m

CR, complete response; PR, partial response; m, month; y, year. ^a^This case had an early-stage disease; ^b^This was a radiological complete response. The plasma EBV-DNA was persistent positive.

**Figure 4:**
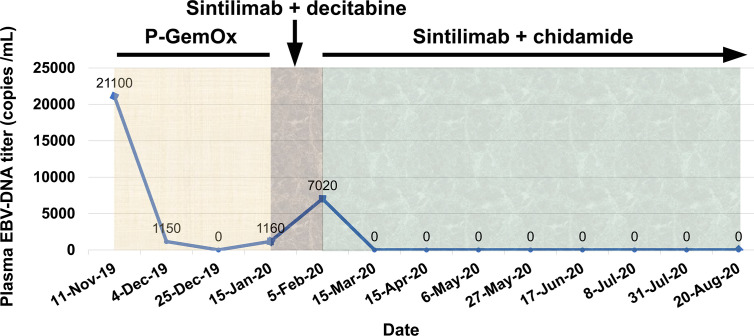
Treatment process and the changes of plasma EBV-DNA titers.

We gave the patient an oral treatment of HDACi chidamide (30 mg every three days) beginning February 5, 2020. Meanwhile, the immunotherapy with sintilimab was administered continuously. The fever disappeared after several days of the combined treatment; the plasma EBV-DNA titer was quickly reduced to undetectable level at the end of the first cycle of the combined treatment and kept at this level thereafter, indicating a molecular complete response (CR) was achieved. A grade 1 thrombocytopenia and anemia occurred during the course of the combined treatment. After four cycles of the combined treatment, the patient’s facial swelling had subsided, and he looked totally normal (**Figure 1F**). The dose of chidamide was reduced to 20 mg every three days thereafter and both thrombocytopenia and anemia were resolved. The patient was very satisfied with the treatment. He went to his work after six cycles of treatment. Hematopoietic stem cell transplantation was recommended in order to improve long-term outcome, but the patient refused. He has received nine cycles of treatment by the submission date of this article. A detailed treatment process and the changes of plasma EBV-DNA titers are shown in **Figure 4**.

## Discussion and Literature Review

According to primary tumor location, NKTCL can be classified into two subtypes (1): nasal NKTCL involving the upper aerodigestive tract, including nasal cavity, nasopharynx, oral cavity, oropharynx, and hypopharynx; and (2) extranasal NKTCL involving any other organ or tissue such as skin, gastrointestinal tract, bone, and lung (18, 19). Extranasal NKTCL accounts for approximately 20% of newly diagnosed cases (20, 21). Generally, the prognosis of extranasal NKTCL is poorer than that of nasal NKTCL. The median overall survival time is only 3.4 months in patients with advanced extranasal NKTCL (19).

Over the past decade, significant progress has been made in the treatment of NKTCL. On the one hand, radiotherapy has been used as a curative option for early-stage NKTCL (22, 23). About 80% early-stage NKTCL patients were cured by radiotherapy alone or in combination with chemotherapy (2). One the other hand, the importance of L-asparaginase has been widely recognized in the treatment of NKTCL. The CR rate generally exceeded 50% in NKTCL patients treated with asparaginase (or pegaspargase)-containing chemotherapy. However, these progresses largely benefited those patients with early-stage disease. Relatively, patients with advanced disease had a dismal outcome, with a median survival of only several months even asparaginase-based chemotherapy was used (3, 21). Besides, asparaginase-based chemotherapy did not seem to improve the survival of patients with extranasal NKTCL (24, 25). Thus, novel treatment strategies are urgently needed for NKTCL patients with advanced disease, especially asparaginase-resistant disease.

NKTCL is universally associated with EBV infection in the lymphoma cells. EBV-DNA levels in peripheral blood are a surrogate biomarker of tumor loads, which are useful for prognostic assessment and treatment response evaluation. High plasma EBV-DNA levels, both pre- and post-treatment, correlate with worse clinical outcomes (26, 27). On the other hand, EBV infection is associated with higher PD-L1 expression in lymphoma cells and better response to immunotherapy (9, 10, 28, 29). Several case reports, case series, and small sample-sized phase 2 trials have demonstrated the anticancer activity of PD-1/PD-L1 blockade immunotherapy in NKTCL patients (**Table 1**). Based on these practices, the PD-1 inhibitors pembrolizumab and nivolumab were recently recommended by the NCCN guidelines as an alternative treatment option for r/r NKTCL. It seems that immunotherapy has convincing short-term efficacy in r/r NKTCL, but the long-term outcomes of immunotherapy-treated patients are still disappointing. Until now, the largest prospective clinical trial involving immunotherapy in r/r NKTCL patients has been conducted in China, in which 28 patients were treated with sintilimab. Of them, 19 patients achieved an objective response (8). In another phase 2 trial enrolling 21 patients in Korea, avelumab achieved CR in five patients and PR in three patients, with a median PFS of 2.7 months (9). Kim et al. reported five out of 14 patients treated with pembrolizumab achieved CR (10). Two retrospective case series studies reported five CR and one CR in seven and three patients, respectively (11, 13). In another case series study with seven patients with pembrolizumab treatment, two patients achieved CR, and the median PFS was 4.8 months (12). Klee et al. reported one patient who had survived more than 2 years following a combined treatment of pembrolizumab and radiotherapy, but it should be noted that this case had an early-stage disease (14). With the emergence of various new anti-tumor drugs, immunotherapy-based treatment for NKTCL is continuously evolving to improve patient outcomes. Clinical trials evaluating the combination of immunotherapy with various cancer therapies are currently under investigation, including cytotoxic drugs, PI3K inhibitor, HDACi, CAR-T cell therapy, and antiangiogenic agent (**Table 2**).

**Table 2:** Ongoing clinical trials utilizing immune checkpoint blockade in NKTCL (by 10-Aug-2020).

Phase	Trial Intervention	Status; Estimated completion date	NCT ID
1	MEDI-570 (anti-ICOS antibody)	Recruiting; December 2020	02520791
1	Pembrolizumab + modified SMILE and ASCT	Recruiting; December 2023	03719105
1/2	Pembrolizumab Pembrolizumab + copanlisib	Recruiting; December 2022	02535247
1/2	Sintilimab + chidamide	Recruiting; February 2025	03820596
1/2	Pembrolizumab + romidepsin	Active, not recruiting; November 2020	03278782
1/2	Pembrolizumab + pralatrexate	Recruiting; April 2021	03598998
2	Nivolumab + talimogene laherparepvec	Recruiting; June 2021	02978625
2	Pembrolizumab + radiotherapy	Recruiting; November 2020	03210662
NA	Cemrelizumab + pegaspargase + apatinib	Recruiting; December 2023	04366128

ICOS, Inducible T-cell co-stimulator; SMILE, Dexamethasone, methotrexate, ifosfamide, pegaspargase, and etoposide; ASCT, Allogeneic stem cell transplantation; NA, not availabe.

Chidamide, a subtype-selective HDACi, was approved in China for patients with relapsed or refractory peripheral T-cell lymphoma. It is also used to treat r/r NKTCL. In a phase 2 trial including various types of T- and NK-cell lymphomas, the ORR was 19% in 16 NKTCL patients and the median PFS was 2.1 months (30). In addition to its direct anticancer activity, HDACi has pleiotropic immunomodulatory effects (31–33). HDACi can enhance the intratumoral infiltration of CD8+ T cells and macrophages, decrease the intratumoral infiltration of T-regulatory cells, myeloid-derived suppressor cells, and pro-tumorigenic M2 macrophages, induce the intratumoral expression of multiple chemokines, upregulate the expression of MHC and co-stimulatory molecules, enhance immune recognition, promote tumor-specific T cell-mediated killing of cancer cells, and sensitize tumor cells to NK cell lysis (32, 34–42). Preclinical studies have demonstrated that HDACi can enhance the anticancer activity of immunotherapy in several types of cancers (36, 37, 43–45). Currently, there are dozens of clinical trials evaluating the feasibility of combining HDACi and immunotherapy across multiple cancer types (32, 34).

In this study, the primary cutaneous NKTCL acquired resistance after a short response to pegaspargase-based chemotherapy. It was known that a transient increase of tumor volume, called pseudoprogression, in immunotherapy-treated patients. Nevertheless, it was unlikely a pseudogprogression in this case after one cycle of sintilimab and decitabine combination therapy, as together with tumor regrowth, the lymphoma-related fever reappeared and the plasma EBV-DNA titer, a sensitive indicator of NKTCL load, was increased following the combined treatment. Therefore, we believe that this case was primarily resistant to sintilimab. Based on the aforementioned immunomodulatory effects of HDACi, chidamide was used in combination with sintilimab as third-line treatment for this patient. An excellent response was observed: the fever and plasma EBV-DNA disappeared very quickly after the first cycle of the combined regimen treatment, and the response has lasted for over 6 months so far. The result of this study should be interpreted with caution, because chidamide alone has anti-cancer activity. It is not sure whether the excellent response in this patient was attributed to the synergistic action of sintilimab and chidamide or to chidamide alone. Currently, there is an ongoing phase 1/2 trial assessing the efficacy and safety of sintilimab plus chidamide in patients with r/r NKTCL (NCT 03820596). It may take time to get the results but it is worth the wait.

## Conclusion

There are limited treatment options for patients with r/r NKTCL, especially those resistant to asparaginase and immunotherapy. We reported herein that a pegaspargase- and immunotherapy-resistant patient achieved durable response from the combined treatment of sintilimab and chidamide with mild toxicity. This combination regimen of immunotherapy and HDACi is a promising treatment choice for patients with r/r NKTCL.

## Data Availability Statement

The original contributions presented in the study are included in the article/supplementary materials. Further inquiries can be directed to the corresponding author.

## Ethics Statement

The studies involving human participants were reviewed and approved by Institutional Review Board of Affiliated Cancer Hospital of Zhengzhou University. The patients/participants provided their written informed consent to participate in this study. Written informed consent was obtained from the individuals for the publication of any potentially identifiable images or data included in this article.

## Author Contributions

ZheY and ZhiY: designed the study. ZheY, SY, YL, HW, JC, SZ, and ZhiY: treated the patient and collected the data. ZheY, JZ, and PL: collected and analyzed the data. ZheY and ZhiY: wrote the original draft. All authors contributed to the article and approved the submitted version.

## Conflict of Interest

The authors declare that the research was conducted in the absence of any commercial or financial relationships that could be construed as a potential conflict of interest.

## References

[1] Aozasa K Zaki MA Epidemiology and pathogenesis of nasal NK/T-cell lymphoma: a mini-review. ScientificWorldJournal (2011) 11:422–8. 10.1100/tsw.2011.41 PMC5720017 21336457

[2] Qi SN Yang Y Zhang YJ Huang HQ Wang Y He X Risk-based, response-adapted therapy for early-stage extranodal nasal-type NK/T-cell lymphoma in the modern chemotherapy era: A China Lymphoma Collaborative Group study. Am J Hematol (2020). 10.1002/ajh.25878 32449800

[3] Kim SJ Park S Kang ES Choi JY Lim DH Ko YH Induction treatment with SMILE and consolidation with autologous stem cell transplantation for newly diagnosed stage IV extranodal natural killer/T-cell lymphoma patients. Ann Hematol (2015) 94(1):71–8. 10.1007/s00277-014-2171-4 25082384

[4] Ando M Sugimoto K Kitoh T Sasaki M Mukai K Ando J Selective apoptosis of natural killer-cell tumours by l-asparaginase. Br J Haematol (2005) 130(6):860–8. 10.1111/j.1365-2141.2005.05694.x 16156856

[5] Pokrovsky VS Vinnikov D L-Asparaginase for newly diagnosed extra-nodal NK/T-cell lymphoma: systematic review and meta-analysis. Expert Rev Anticancer Ther (2017) 17(8):759–68. 10.1080/14737140.2017.1344100 28621166

[6] Wang L Li LR Zhang L Wang JW The landscape of new drugs in extranodal NK/T-cell lymphoma. Cancer Treat Rev (2020) 89:102065. 10.1016/j.ctrv.2020.102065 32653806

[7] Nie J Wang C Liu Y Yang Q Mei Q Dong L Addition of Low-Dose Decitabine to Anti-PD-1 Antibody Camrelizumab in Relapsed/Refractory Classical Hodgkin Lymphoma. J Clin Oncol (2019) 37: (17):1479–89. 10.1200/JCO.18.02151 31039052

[8] Tao R Sintilimab for relapsed/refractory (r/r) extranodal NK/T cell lymphoma (ENKTL): A multicenter s-a, phase 2 trial (ORIENT-4). Chicago, IL: John Wiley and Sons (2019) p. 102–3. 10.1038/s41392-021-00768-0 PMC8548511 34702811

[9] Kim SJ Lim JQ Laurensia Y Cho J Yoon SE Lee JY Avelumab for the treatment of relapsed or refractory extranodal NK/T-cell lymphoma: an open-label phase 2 study. Blood (2020) 113:3931–7. 10.1182/blood.2020007247 32766875

[10] Kim SJ Hyeon J Cho I Ko YH Kim WS Comparison of Efficacy of Pembrolizumab between Epstein-Barr VirusPositive and Negative Relapsed or Refractory Non-Hodgkin Lymphomas. Cancer Res Treat (2019) 51(2):611–22. 10.4143/crt.2018.191 PMC6473267 30025443

[11] Kwong YL Chan TSY Tan D Kim SJ Poon LM Mow B PD1 blockade with pembrolizumab is highly effective in relapsed or refractory NK/T-cell lymphoma failing l-asparaginase. Blood (2017) 129(17):2437–42. 10.1182/blood-2016-12-756841 28188133

[12] Li X Cheng Y Zhang M Yan J Li L Fu X Activity of pembrolizumab in relapsed/refractory NK/T-cell lymphoma. J Hematol Oncol (2018) 11(1):15. 10.1186/s13045-018-0559-7 29386072 PMC5793390

[13] Chan TSY Li J Loong F Khong PL Tse E Kwong YL PD1 blockade with low-dose nivolumab in NK/T cell lymphoma failing L-asparaginase: efficacy and safety. Ann Hematol (2018) 97(1):193–6. 10.1007/s00277-017-3127-2 28879531

[14] Klee G von Ducker L Terheyden P Sustained complete remission of extranodal NK/T-cell lymphoma, nasal type, following pembrolizumab and radiation therapy. J Dtsch Dermatol Ges (2020) 98:1647–55. 10.1111/ddg.14142 32578307

[15] Kim YE Kim H Shin J Min SY Kang SH Suh JK Stage IV natural killer/T-cell lymphoma with chronic active Epstein-Barr virus, treated with pembrolizumab and TCRalphabeta-depleted haploidentical hematopoietic stem cell transplantation. Leuk Lymphoma (2020) 1-4:106284. 10.1080/10428194.2020.1757666 32352338

[16] Asif S Begemann M Bennett J Fatima R Masood A Raza S Pembrolizumab in newly diagnosed EBV-negative extranodal natural killer/T-cell lymphoma: A case report. Mol Clin Oncol (2019) 10(3):397–400. 10.3892/mco.2019.1805 30847181 PMC6388498

[17] Lai J Xu P Jiang X Zhou S Liu A Successful treatment with anti-programmed-death-1 antibody in a relapsed natural killer/T-cell lymphoma patient with multi-line resistance: a case report. BMC Cancer (2017) 17(1):507. 10.1186/s12885-017-3501-4 28754096 PMC5534108

[18] Jo JC Yoon DH Kim S Lee BJ Jang YJ Park CS Clinical features and prognostic model for extranasal NK/T-cell lymphoma. Eur J Haematol (2012) 89(2):103–10. 10.1111/j.1600-0609.2012.01796.x 22553935

[19] Au WY Weisenburger DD Intragumtornchai T Nakamura S Kim WS Sng I Clinical differences between nasal and extranasal natural killer/T-cell lymphoma: a study of 136 cases from the International Peripheral T-Cell Lymphoma Project. Blood (2009) 113(17):3931–7. 10.1182/blood-2008-10-185256 19029440

[20] Suzuki R Suzumiya J Yamaguchi M Nakamura S Kameoka J Kojima H Prognostic factors for mature natural killer (NK) cell neoplasms: aggressive NK cell leukemia and extranodal NK cell lymphoma, nasal type. Ann Oncol (2010) 21(5):1032–40. 10.1093/annonc/mdp418 19850638

[21] Yan Z Huang HQ Wang XX Gao Y Zhang YJ Bai B A TNM Staging System for Nasal NK/T-Cell Lymphoma. PLoS One (2015) 10(6):e0130984. 10.1371/journal.pone.0130984 26098892 PMC4476596

[22] Deng XW Wu JX Wu T Zhu SY Shi M Su H Radiotherapy is essential after complete response to asparaginase-containing chemotherapy in early-stage extranodal nasal-type NK/T-cell lymphoma: A multicenter study from the China Lymphoma Collaborative Group (CLCG). Radiother Oncol (2018) 129(1):3–9. 10.1016/j.radonc.2018.04.026 29739712

[23] Vargo JA Patel A Glaser SM Balasubramani GK Farah RJ Marks SM The impact of the omission or inadequate dosing of radiotherapy in extranodal natural killer T-cell lymphoma, nasal type, in the United States. Cancer (2017) 123(16):3176–85. 10.1002/cncr.30697 28380259

[24] Yamaguchi M Suzuki R Miyazaki K Amaki J Takizawa J Sekiguchi N Improved prognosis of extranodal NK/T cell lymphoma, nasal type of nasal origin but not extranasal origin. Ann Hematol (2019) 98(7):1647–55. 10.1007/s00277-019-03689-9 31001658

[25] Jiang L Li P Quan Q Chen P Qiu H Zhang B Cutaneous extranodal natural killer (NK) / T - cell lymphoma: A comprehensive clinical features and outcomes analysis of 71 cases. Leuk Res (2020) 88:106284. 10.1016/j.leukres.2019.106284 31841860

[26] Suzuki R Yamaguchi M Izutsu K Yamamoto G Takada K Harabuchi Y Prospective measurement of Epstein-Barr virus-DNA in plasma and peripheral blood mononuclear cells of extranodal NK/T-cell lymphoma, nasal type. Blood (2011) 118(23):6018–22. 10.1182/blood-2011-05-354142 21984805

[27] Kimura H Kwong YL EBV Viral Loads in Diagnosis, Monitoring, and Response Assessment. Front Oncol (2019) 9:62. 10.3389/fonc.2019.00062 30809508 PMC6379266

[28] Bi XW Wang H Zhang WW Wang JH Liu WJ Xia ZJ PD-L1 is upregulated by EBV-driven LMP1 through NF-kappaB pathway and correlates with poor prognosis in natural killer/T-cell lymphoma. J Hematol Oncol (2016) 9(1):109. 10.1186/s13045-016-0341-7 27737703 PMC5064887

[29] Chen BJ Chapuy B Ouyang J Sun HH Roemer MG Xu ML PD-L1 expression is characteristic of a subset of aggressive B-cell lymphomas and virus-associated malignancies. Clin Cancer Res (2013) 19(13):3462–73. 10.1158/1078-0432.CCR-13-0855 PMC4102335 23674495

[30] Shi Y Dong M Hong X Zhang W Feng J Zhu J Results from a multicenter, open-label, pivotal phase II study of chidamide in relapsed or refractory peripheral T-cell lymphoma. Ann Oncol (2015) 26(8):1766–71. 10.1093/annonc/mdv237 26105599

[31] Zhao LM Zhang JH Histone Deacetylase Inhibitors in Tumor Immunotherapy. Curr Med Chem (2019) 26(17):2990–3008. 10.2174/0929867324666170801102124 28762309

[32] Mazzone R Zwergel C Mai A Valente S Epi-drugs in combination with immunotherapy: a new avenue to improve anticancer efficacy. Clin Epigenet (2017) 9:59. 10.1186/s13148-017-0358-y PMC5450222 28572863

[33] Conte M De Palma R Altucci L HDAC inhibitors as epigenetic regulators for cancer immunotherapy. Int J Biochem Cell Biol (2018) 98:65–74. 10.1016/j.biocel.2018.03.004 29535070

[34] Briere D Sudhakar N Woods DM Hallin J Engstrom LD Aranda R The class I/IV HDAC inhibitor mocetinostat increases tumor antigen presentation, decreases immune suppressive cell types and augments checkpoint inhibitor therapy. Cancer Immunol Immunother (2018) 67(3):381–92. 10.1007/s00262-017-2091-y PMC11028326 29124315

[35] Hicks KC Fantini M Donahue RN Schwab A Knudson KM Tritsch SR Epigenetic priming of both tumor and NK cells augments antibody-dependent cellular cytotoxicity elicited by the anti-PD-L1 antibody avelumab against multiple carcinoma cell types. Oncoimmunology (2018) 7(11):e1466018. 10.1080/2162402X.2018.1466018 30377559 PMC6205056

[36] Bretz AC Parnitzke U Kronthaler K Dreker T Bartz R Hermann F Domatinostat favors the immunotherapy response by modulating the tumor immune microenvironment (TIME). J Immunother Cancer (2019) 7(1):294. 10.1186/s40425-019-0745-3 31703604 PMC6839078

[37] Knox T Sahakian E Banik D Hadley M Palmer E Noonepalle S Selective HDAC6 inhibitors improve anti-PD-1 immune checkpoint blockade therapy by decreasing the anti-inflammatory phenotype of macrophages and down-regulation of immunosuppressive proteins in tumor cells. Sci Rep (2019) 9(1):6136. 10.1038/s41598-019-42237-3 30992475 PMC6467894

[38] Ugurel S Spassova I Wohlfarth J Drusio C Cherouny A Melior A MHC class-I downregulation in PD-1/PD-L1 inhibitor refractory Merkel cell carcinoma and its potential reversal by histone deacetylase inhibition: a case series. Cancer Immunol Immunother (2019) 68(6):983–90. 10.1007/s00262-019-02341-9 PMC11028125 30993371

[39] Adeshakin AO Yan D Zhang M Wang L Adeshakin FO Liu W Blockade of myeloid-derived suppressor cell function by valproic acid enhanced anti-PD-L1 tumor immunotherapy. Biochem Biophys Res Commun (2020) 522(3):604–11. 10.1016/j.bbrc.2019.11.155 31785814

[40] Kim YD Park SM Ha HC Lee AR Won H Cha H HDAC Inhibitor, CG-745, Enhances the Anti-Cancer Effect of Anti-PD-1 Immune Checkpoint Inhibitor by Modulation of the Immune Microenvironment. J Cancer (2020) 11(14):4059–72. 10.7150/jca.44622 PMC7196255 32368288

[41] Wang X Waschke BC Woolaver RA Chen SMY Chen Z Wang JH HDAC inhibitors overcome immunotherapy resistance in B-cell lymphoma. Protein Cell (2020) 11(7):472–82. 10.1007/s13238-020-00694-x PMC7305292 32162275

[42] Yeon M Kim Y Jung HS Jeoung D Histone Deacetylase Inhibitors to Overcome Resistance to Targeted and Immuno Therapy in Metastatic Melanoma. Front Cell Dev Biol (2020) 8:486. 10.3389/fcell.2020.00486 32626712 PMC7311641

[43] Terranova-Barberio M Thomas S Ali N Pawlowska N Park J Krings G HDAC inhibition potentiates immunotherapy in triple negative breast cancer. Oncotarget (2017) 8(69):114156–72. 10.18632/oncotarget.23169 PMC5768393 29371976

[44] Llopiz D Ruiz M Villanueva L Iglesias T Silva L Egea J Enhanced anti-tumor efficacy of checkpoint inhibitors in combination with the histone deacetylase inhibitor Belinostat in a murine hepatocellular carcinoma model. Cancer Immunol Immunother (2019) 68(3):379–93. 10.1007/s00262-018-2283-0 PMC11028337 30547218

[45] Burke B Eden C Perez C Belshoff A Hart S Plaza-Rojas L Inhibition of Histone Deacetylase (HDAC) Enhances Checkpoint Blockade Efficacy by Rendering Bladder Cancer Cells Visible for T Cell-Mediated Destruction. Front Oncol (2020) 10:699. 10.3389/fonc.2020.00699 32500025 PMC7243798

